# Delirium in acute promyelocytic leukemia patients: two case reports

**DOI:** 10.1186/1756-0500-6-469

**Published:** 2013-11-16

**Authors:** Gian Matteo Rigolin, Sara Martinelli, Luca Formigaro, Francesca Cibien, Enrico Lista, Maurizio Cavallari, Marco Ambrosio, Miriam Pizzolato, Giulia Daghia, Olga Sofritti, Antonio Cuneo

**Affiliations:** 1 Unità operativa di Ematologia, Dipartimento Medico Specialistico, Azienda Ospedaliero-Universitaria Arcispedale S. Anna, via Aldo Moro, 8-44124, Cona Ferrara, Italy

**Keywords:** Acute promyelocytic leukemia, Delirium, ATRA

## Abstract

**Background:**

Delirium is a frequently misdiagnosed and inadequately treated neuropsychiatric complication most commonly observed in terminally ill cancer patients. To our knowledge this is the first report describing delirium in two patients aged less than 60 years and enrolled in an intensive chemotherapeutic protocol for acute promyelocytic leukemia.

**Case presentation:**

Two female Caucasian acute promyelocytic leukemia patients aged 46 and 56 years developed delirium during their induction treatment with all-trans retinoic acid and idarubicin. In both cases symptoms were initially attributed to all-trans retinoic acid that was therefore immediately suspended. In these two patients several situations may have contribute to the delirium: in patient 1 a previous psychiatric disorder, concomitant treatments with steroids and benzodiazepines, a severe infection and central nervous system bleeding while in patient 2 steroid treatment and isolation. In patient 1 delirium was treated with short-term low-doses of haloperidol while in patient 2 non-pharmacologic interventions had a beneficial role. When the diagnosis of delirium was clear, induction treatment was resumed and both patients completed their therapeutic program without any relapse of the psychiatric symptoms. Both patients are alive and in complete remission as far as their leukemia is concerned.

**Conclusions:**

We suggest that patients with acute promyelocytic leukemia eligible to intensive chemotherapy should be carefully evaluated by a multisciplinary team including psychiatrists in order to early recognize symptoms of delirium and avoid inadequate treatments. In case of delirium, both pharmacologic and non-pharmacologic interventions may be considered.

## Background

Delirium is a disturbance of consciousness characterized by changes in cognition, perceptual disturbances and a reduced ability to focus, sustain, or shift attention. These symptoms occur over a short period of time and tend to fluctuate during the course of the day [1]. Delirium is the most frequent neuropsychiatric complication in terminally ill cancer patients with a prevalence of up to 85% in the last weeks of life [2]. Even though delirium is associated with a significant morbidity and mortality, it is frequently underdiagnosed or misdiagnosed and therefore inadequately treated [2].

In hematologic patients, delirium has been mainly observed in elderly cases not eligible for intensive chemotherapeutic regimens [3] and in subjects undergoing myeloablative hematopoietic stem-cell transplantation, with a prevalence of nearly 50% during the 4 weeks after conditioning and stem-cell infusion [4].

In adults, acute promyelocytic leukemia (APL) is the most curable subtype of acute myeloid leukemias. APL is frequently considered a medical emergency because of its life threatening coagulation complications [5] that require immediate treatment with both all-trans retinoic acid (ATRA)-based therapies and measures to counteract the coagulopathy [6].

In this report we present two APL patients aged less than 60 years who developed delirium in the early phases of their induction treatment with ATRA and idarubicin. In both cases, the symptoms were initially attributed to ATRA treatment that was interrupted. However subsequently, in both cases a diagnosis of delirium was made and the treatment for APL was therefore resumed along with an intervention for the psychiatric disorder.

## Case presentation

### Case 1

Misses G, a 46-year-old married Caucasian woman, was admitted in October 2010 to our hematology unit for bleeding signs (petecchiae, hematomas), pancytopenia (white blood cell count 0.24 × 10^3^/μL, hemoglobin 6.6 g/dL, platelet count 35 × 10^3^/μL) with normal prothrombin time (PT) and activated partial thromboplastin time (APTT) coagulation tests but a slightly reduced fibrinogen level (113 mg/dL) and increased D-Dimer (2017 ng/mL: normal value < 370). In adolescence she had a history of eating disorder. Bone marrow aspirate showed 90% of hypergranular blasts expressing the following markers: myeloperoxidase, CD13 and CD33. CD34 and HLA-DR were instead not expressed on blast cells. Cytogenetic analysis showed the classical translocation t(15;17)(q22;q21) [7]. A diagnosis of APL was therefore made. At diagnosis computed tomography (CT) and magnetic resonance imaging (MRI) scans showed the presence of 2 small hemorrhagic lesions in the suprachiasmatic region and in the right cerebellum. Neurologic examination was normal. The patient immediately started an induction therapy with ATRA (45 mg/m^2^/day) and idarubicin (12 mg/m^2^ on days 2-4-6-8). Prednisone (0.5 mg/kg/day) was given to prevent differentiation syndrome [6]. After 7 days of treatment the patient exhibited difficulty in focusing attention, anxiety, hyperpnoea, restlessness and irritability. In the suspect of differentiation syndrome, ATRA was suspended, while steroids were administered at therapeutic dosage (dexamethasone 8 mg intravenous every 12 hours) along with a concomitant treatment with oral benzodiazepine for the state of anxiety. In the following 10 days the patient showed a progressive worsening of her psychological status with disorientation, disorganized thinking, misperceptions, hallucinations, severe neurological abnormalities including tremor, dysgraphia, apraxia, aphasia. At this point, according to the Diagnostic and Statistical Manual of Mental Disorders, 4th Edition (DSM-IV) criteria as previously described [8], a diagnosis of mixed hyperactive and hypoactive delirium was made and treatment with haloperidol (0.5 mg per os or intravenous every 12 hours) was started, while lorazepam 0.5 mg intravenous was used in case of agitation. ATRA administration was resumed. In the following days the patient worsened because of sepsis by *Enterecoccus faecalis* and subsequent pneumonitis that required antibiotics. The clinical course of the delirium showed a consensual worsening characterized by profound lethargy with an Eastern Cooperative Oncology Group (ECOG) performance status of 4 (totally confined to bed) requiring parenteral feeding and supportive care. After 30 days from the beginning of induction treatment the patient showed hematologic recovery which anticipated a progressive improvement of her clinical and psychiatric picture. After 25 days of treatment haloperidol was stopped. A rehabilitative program was begun and she was finally dismissed in complete hematologic response after 64 days. She was subsequently readmitted to our unit to complete, without further significant clinical problems, the therapeutic protocol which included 3 consolidation chemotherapeutic regimens. The patient is now alive and well, in complete remission after 2 years from diagnosis.

### Case 2

Misses S, a 56-year-old married Caucasian woman, was admitted in July 2012 to our hematology unit for bleeding signs (petecchiae, hematomas), pancytopenia (white blood cell count 0.55 × 10^3^/μL, hemoglobin 7.6 g/dL, platelet count 47 × 10^3^/μL) with normal fibrinogen level and APTT, increased PT (1.46 INR), and increased D-Dimer (7496 ng/mL: normal value < 370). Bone marrow aspirate showed 95% of hypergranular myeloperoxidase positive, CD13 positive, CD33 negative, HLA-DR negative and CD34 negative blasts with cytogenetic analysis demonstrating the translocation t(15;17)(q22;q21), thus confirming the diagnosis of APL. The patient immediately started induction therapy including ATRA (45 mg/m^2^/day for 28 days) and idarubicin (12 mg/m^2^ on days 2-4-6-8). Prednisone (0.5 mg/kg/day) was given to prevent differentiation syndrome. During this period the patient was in isolation in a positive-pressure ventilated room. After 8 days of treatment the patient showed agitation, euphoria, altered perceptions including misperceptions, illusions, acoustic hallucinations. ATRA was temporarily suspended and steroids were administered at higher levels (dexamethasone 8 mg intravenous every 12 hours). Since CT scan, lumbar puncture and audiological evaluation were all negative, a diagnosis of hyperactive delirium was made according to DSM-IV criteria as previously described [8]. After 4 days of suspension, ATRA treatment was therefore resumed, steroids were tapered, while for her delirium non-pharmacologic interventions were adopted including environmental family support and reallocation in a double room. Thereafter the patient progressively recovered from her psychological status, completed induction treatment. She was finally dismissed after 35 days in complete hematologic response with a complete recovery from the psychiatric symptoms. The patient subsequently completed 3 consolidation courses of chemotherapy and is now in complete remission. No relapse of delirium was observed in the subsequent readmissions to the clinic.

## Conclusions

In advanced cancer patients, delirium is often associated with a poor prognosis, although 50% of patients can improve with appropriate management of contributing etiologies. Early recognition of delirium and treatment are therefore important in diminishing morbidity and mortality [2].

In patients with cancer, delirium can result either from a direct effect of the cancer on the central nervous system (CNS) or from indirect CNS effects related to the clinical course of the disease (ie, older age, electrolyte imbalance, dehydration, major organ failures, infections, vascular complications, para-neoplastic syndromes, border line cognitive impairment) [9]. Chemotherapeutic and immunotherapeutic agents (i.e.; vincristine, corticosteroids, interferon) and medications used in supportive care (i.e., opioids, benzodiazepines, hypnotics, antidepressant, antiemetics) may contribute to delirium in these patients [10]. According to the level of psychomotor activity, three clinical delirium subtypes have been described: hypoactive, hyperactive, and mixed (with alternating features of both hyperactive and hypoactive delirium) [2]. The hypoactive subtype is characterized by psychomotor retardation, lethargy, and reduced awareness of surroundings. The hyperactive subtype is commonly characterized by restlessness, agitation, hyper-vigilance, hallucinations, and delusions. Hypoactive delirium is often mistaken for depression and is difficult to differentiate from pharmacological sedation, or obtundation in the last days of life.

To our knowledge this is the first report describing delirium in APL patients aged less than 60 years and enrolled in an intensive chemotherapeutic regimen. In our two cases several situations that could have precipitated the psychiatric status were present: in patient 1 a previous psychiatric disorder, concomitant treatments with steroids and benzodiazepines, a severe infection and CNS bleeding while in patient 2 we could annotate steroid treatment and isolation. It is important that clinicians include delirium as a possible complication in the treatment of APL patients eligible for intensive regimens and that they consider concomitant clinical conditions or medications that may precipitate this condition. This is critical in order to avoid a misdiagnosis that may lead to the suspension of APL treatment resulting in a reduced response rate. However, psychiatrists asked to consult on patients being treated for APL should also be aware that the symptoms of differentiation syndrome, a complication occurring in up to 25% of these cases, may also be misinterpreted as a primary psychiatric disorder [11].

In cancer patients with delirium both pharmacologic and non-pharmacologic interventions can be used [9]. Pharmacologic interventions include short-term low-dose of antipsychotics, with close monitoring for possible adverse effects, particularly in patients with multiple medical comorbidities. However, non-pharmacologic interventions also appear to have a beneficial role in the treatment of cancer patients with delirium. Based on the evidence from non-oncology settings, it is strongly recommended to implement non-pharmacologic interventions in patients with delirium or at risk for delirium since no serious complications have been observed with a non-pharmacologic strategy [9].

In conclusion, we believe that patients with APL eligible for intensive chemotherapeutic regimens should be carefully evaluated by a multisciplinary team including psychiatrists in order to early recognize delirium symptoms and avoid inadequate treatments. In case of delirium, both pharmacologic and non-pharmacologic interventions may be considered.

## Consent

Written informed consent was obtained from the two patients for publication of this case report. A copy of the written consent is available for review by the Editor-in-Chief of this journal.

## Competing interests

The authors declare that they have no competing interests.

## Authors’ contributions

GMR and AC designed the study, analysed clinical data and wrote the paper. GMR, SM, LF, FC, EL, MC, GD, OS took care of the patients and aided in preparing the manuscript. All authors approved the submitted version of the manuscript
